# When memory fades, meaning remains: Personhood and palliative presence in dementia care

**DOI:** 10.1017/S147895152510062X

**Published:** 2025-09-12

**Authors:** Jose Eric Mella Lacsa

**Affiliations:** Department of Theology and Religious Education, De La Salle University, Manila, Philippines

Dear Editor,

The global rise in dementia cases has pushed palliative care to reimagine its scope, especially for conditions with high symptom burden and psychosocial complexity. A recent longitudinal narrative study by Bentley et al. (2025) in the UK provides vital insights into the lived experiences of individuals with Lewy body dementia (LBD), a neurodegenerative disease characterized by severe cognitive fluctuations, visual hallucinations, and Parkinsonian features. What sets this study apart is not only its focus on an understudied dementia subtype but its methodological depth: by following 5 couples over time, it humanizes the profound intersection of suffering, caregiving, and clinical abandonment in LBD.

The study foregrounds the relational losses endured, communication breakdown, declining continence, and diminishing energy, which cumulatively erode independence. Yet, the most wrenching insight comes from caregivers, who describe a “slow disappearance” of companionship. This emotional toll, exacerbated by insufficient clinical recognition and support, often leaves families feeling isolated in the care journey. Importantly, Bentley et al. apply Murray’s (2000) multi-level narrative analysis to situate these personal losses within broader interpersonal, systemic, and cultural frames, thereby offering a textured and globally resonant portrait of how LBD unravels lives across time.

What emerges is a profound tension in current dementia discourse. While global health agendas now emphasize “living well with dementia” (Quinn et al. 2022), this study critiques the unintended silencing of legitimate grief, decline, and loss, particularly in diseases like LBD, where aggressive symptom progression resists facile optimism. The authors urge a palliative care lens that does not negate hope, but reframes it: one that accompanies decline with dignity, validates suffering, and advocates for better continuity of care.

This work carries significant global implications. In low-resource settings, where formal dementia services are scarce, the call for early integration of palliative principles, symptom control, psychosocial support, and anticipatory guidance, is both urgent and actionable (Prince et al. 2011). Bentley et al. highlight that such care need not await terminal stages; it can and must be embedded throughout the disease trajectory.

As the world grapples with aging populations and rising dementia rates, the LBD experience offers a sobering reminder: that clinical authority must be reoriented not only around diagnostics, but around listening deeply to the lives unfolding in its shadow. Person-centered, narrative-informed palliative care is no longer optional, it is a moral imperative.
